# Ampelopsis Grossedentata Flavonoids Alleviate Hepatic Damage Through Remodeling Intestinal Microflora and Reducing Oxidative Stress in Rats

**DOI:** 10.1002/fsn3.71746

**Published:** 2026-04-20

**Authors:** Hao Shi, Bo Zhou, Jing Wang, Li Lan Lei

**Affiliations:** ^1^ College of Life and Environmental Sciences Hunan University of Arts and Science Changde Hunan China; ^2^ College of Agriculture and Forestry Science Hunan Applied Technology University Changde Hunan China

**Keywords:** Ampelopsis grossedentata flavonoids, antioxidant activity, gut microbiota, immunoregulation, rats

## Abstract

To investigate the antioxidant effects and underlying mechanisms of *Ampelopsis grossedentata* flavonoids (AGF) in rats fed a high‐fat diet. In this study, we established a basal diet group, a high‐fat diet group, a high‐fat diet combined with Xuezhikang group, and high‐fat diet groups supplemented with low, medium, and high doses of AGF. After 7 weeks of feeding, relevant physiological antioxidant indicators and the antioxidant effects mediated by gut microbiota were measured. The results demonstrated that: (1) AGF significantly increased the gene expression, protein levels, and enzymatic activity of CAT and SOD in the livers of rats fed a high‐fat diet, thereby increasing the total antioxidant capacity and reducing power in serum. (2) Simultaneously, AGF demonstrated strong free radical (DPPH, hydroxyl, superoxide anion, and ABTS) scavenging capacity, accompanied by a reduction in hydrogen peroxide content and anti‐lipid peroxidation activity. (3) Furthermore, AGF significantly decreased the levels of pro‐inflammatory cytokines (IL‐6, IL‐1β, TNF‐α) in the livers of high‐fat diet‐fed rats. (4) Additionally, flavonoids enriched the diversity of gut microbiota, optimized microbial structure, and increased the abundance of beneficial microbes (such as *Akkermansia*, *Lactobacillus*, *Blautia*, *norank_f__Eubacterium_coprostanoligenes_group*, *Enterorhabdus*, *Butyricicoccus*, and *Clostridium_innocuum_group*), which showed positive correlations with antioxidant‐related physiological parameters. In conclusion, AGF significantly alleviated high‐fat diet‐induced oxidative stress and liver injury in rats, providing a theoretical foundation for developing H‐AGF as a functional ingredient for use in functional foods, pharmaceuticals, and skincare products.

## Graphical abstract

This graphical abstract illustrates the multi‐target antioxidant and anti‐inflammatory effects of AGF in alleviating liver injury, and their association with gut microbiota modulation and enrichment of beneficial microbes.

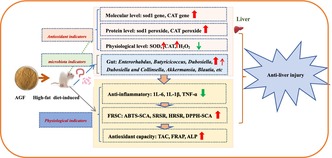

**Abbreviations**
ABTS‐SCA: ABTS scavenging capacity
ALP: Anti‐lipid peroxidation
DPPH‐SCA: DPPH scavenging capacity
FRAP: Ferric ion reducing antioxidant
FRSC: Free Radical Scavenging Capacity
HRSR: Hydroxyl radical scavenging rate
SRSR: Superoxide radical scavenging rate
T‐AOC: Total antioxidant capacity

## 1 Introduction

Vine tea, also known as *Ampelopsis grossedentata*, is a deciduous liana species belonging to the Vitaceae family. It is primarily distributed in the Xiangxi region of China and has a cultivation history spanning over 1200 years (Zeng et al. 2023; Wang et al. 2024). As a tea beverage, vine tea enjoys high popularity due to its unique nutritional and health benefits (such as antioxidant, anti‐aging, and anti‐inflammatory effects, lipid‐lowering, liver protection, regulation of gut health, improvement of digestive function, etc.) and its high economic value (Wu et al. 2023). Vine tea is rich in a variety of chemical components, including flavonoids, polyphenols, sterols, terpenes, polysaccharides, amino acids, volatile oils, and trace elements. Its total flavonoid content exceeds 30%. The primary flavonoids in vine tea are dihydromyricetin, myricetin, quercetin, and kaempferol, among which dihydromyricetin is the most abundant, constituting approximately 80% of the total flavonoids (Carneiro et al. 2021; Zheng et al. 2014). Gao found that dihydromyricetin can treat carbon tetrachloride‐induced liver fibrosis in mice by reducing the expression of pro‐inflammatory cytokines and fibrosis marker mRNA, restoring the richness and diversity of the gut microbiota, and optimizing its structure (Gao et al. 2025). Teng revealed that dihydromyricetin can protect against cyclophosphamide‐induced hepatotoxicity in mice by increasing the activities of superoxide dismutase and catalase, alleviating oxidative stress, and regulating energy metabolism (Teng and Wang 2025). Lv found that dihydromyricetin could alleviate hepatic fat deposition in obese mice induced by a high‐fat diet, with its mechanisms related to the suppression of oxidative stress, inflammation, and lipid synthesis, as well as the promotion of lipid breakdown (Lv et al. 2022). In addition, Lyu revealed that casein/cyanidin‐3‐O‐glucoside and dihydromyricetin could alleviate oxidative stress induced by a high‐fat diet and achieve liver protection by modifying the intestinal microbial composition in mice (increasing the abundance of beneficial microbes while reducing pathogenic ones) (Lyu et al. 2022, 2023). With the gradual improvement of people's living standards, the excessive intake of high‐fat, high‐calorie, and high‐protein foods can generate a large amount of free radicals during digestion, increasing the burden on liver function, damaging liver cells, and easily leading to diseases such as hepatitis, fatty liver disease, and cirrhosis (Vilela et al. 2021). Therefore, the development of natural flavonoid‐based antioxidants for hepatoprotective foods and pharmaceuticals has been drawing increasing attention. In our previous study on the extraction and purification processes of Ampelopsis grossedentata and its antioxidant capacity, we found that it contains high levels of flavonoids and demonstrates potent antioxidant activity (Shi et al. 2025). However, no systematic studies have been reported on the purified flavonoids from vine tea regarding their in vivo antioxidant effects, lipid‐lowering properties, and impacts mediated by intestinal microbiota. Therefore, this study administered vine tea flavonoids to high‐fat diet rats and employed an integrated approach combining physiological indicators, transcriptomics, and microbiomics to comprehensively evaluate their in vivo antioxidant activity and lipid‐lowering potential and efficacy. The aim is to provide a theoretical foundation for developing vine tea flavonoids into functional products with lipid‐lowering activity.

## 2 Materials and Methods

### 2.1 Materials

Xuezhikang (XZK), a red yeast rice extract containing a combination of lovastatin, phytosterols, and isoflavones, was purchased from Yifeng Pharmacy (Changde City, Hunan Province, China). Five‐week‐old Sprague Dawley (SD) rats of SPF grade were purchased from Shulaibao (Wuhan) Biotechnology Co. Ltd., Wuhan, China. License No. SCXK(Xiang) 2022–0011. High‐fat feed (D12492) and basic feed were purchased from Suzhou Shuangshi Experimental Animal Stall Food Technology Co. Ltd., Suzhou, China. The formula of the basic diet per 1000 g was as follows: 390 g corn meal, 200 g wheat flour, 20 g soybean oil, 250 g soybean meal (43% crude protein), 50 g fish meal (67% crude protein), 10 g dextrin, 50 g rat premix, and 30 g alfalfa meal.

### 2.2 Feeding of Rats

The rats were given an adaptation period (Basic diet) for 5 days, and then 36 healthy rats were randomly divided into 6 groups. Rats in each group were housed in individual cages and fed either a basal (Control) or a high‐fat diet (Model) (Table 1). The feeding conditions were as follows: the temperature was maintained between 20°C and 30°C with a humidity of 40%–70%. There was a 12‐h cycle of alternating light and darkness. The animals had free access to food and water. The bedding was changed every 3 days, and the cages were cleaned and sterilized weekly. Rats were orally administered XZK (Positive control) and different concentrations of AGF once a day around 9:30 am, and the rats were allowed to drink and eat freely the rest of the day for 7 weeks. After the last dose, the rats were fasted for 12 h. Blood was then taken by removing the eyeballs, and enzyme activity (serum) and gene and protein levels were measured (liver). All protocols in this study were approved by the ethical committee (reference number JSDX‐2024‐01).

**TABLE 1:** Different feeding treatments of rats.

Group	Diet	Treatment
Control (A)	Basal diet	Saline
Model (B)	High‐fat diet	Saline
L‐AGF	High‐fat diet	50 mg·kg^−1^ (BW) AGF
M‐AGF	High‐fat diet	100 mg·kg^−1^ (BW) AGF
H‐AGF (D)	High‐fat diet	200 mg·kg^−1^ (BW) AGF
Positive (C)	High‐fat diet	60 mg·kg^−1^ (BW)XZK

### 2.3 Total Antioxidant Capacity (T‐AOC) and Ferric Ion Reducing Antioxidant (FRAP) Power Determination

Determination of total antioxidant activity. Transfer 1 mL of the serum and 3.0 mL of a molybdenum phosphate reagent solution (containing 0.6 mol/L sulfuric acid, 28 mmol/L sodium phosphate, and 4 mmol/L ammonium molybdate) into a test tube. Then, place the test tube in a 95°C water bath and let it react for 90 min. After cooling to room temperature, measure the absorbance at a wavelength of 695 nm. A higher absorbance value indicates a stronger total oxidative capacity.

Determination of Ferric Ion Reducing Power. Add 1 mL of the serum, 1 mL of PBS buffer solution, and 1 mL of 1.0% K_3_Fe(CN)_6_ solution into a test tube. Then, place the test tube in a 50°C water bath for 30 min. After cooling to room temperature, add 1 mL of 10% trichloroacetic acid solution and 1 mL of 0.1% FeCl_3_ **·**6H_2_O (3.70 mmol/L Fe^3+^) solution. After 10 min, measure the absorbance at a wavelength of 700 nm. A higher absorbance value indicates a stronger total oxidative capacity.

### 2.4 Determination the Free Radical Scavenging Rate of Hydroxyl, DPPH, Superoxide Anion, and ABTS

Scavenging activity against DPPH radicals. A volume of 3 mL of 0.08 mmol/L DPPH working solution was mixed with 1 mL of the serum in a test tube. The mixture was allowed to react at room temperature for 30 min, and the absorbance was then measured at 517 nm.

Scavenging activity against superoxide anion radicals. A mixture of 0.1 mL of 7.93 mmol/L pyrogallol solution, 5.7 mL of PBS buffer (pH 8.2), and 0.2 mL of the serum was prepared in a test tube. After reaction for 4 min, the absorbance was measured at 320 nm.

Scavenging activity against hydroxyl radicals. A volume of 2 mL of 6 mmol/L ferrous sulfate solution, 2 mL of the serum, and 2 mL of 6 mmol/L hydrogen peroxide were combined in a test tube and mixed thoroughly. After reacting for 10 min, 2 mL of 6 mmol/L salicylic acid solution was added, and the reaction was allowed to proceed for an additional 10 min. The absorbance was finally measured at 510 nm.

Scavenging activity against ABTS cation radicals. The ABTS cation radical working solution was prepared by mixing 5.00 mL of 7 mmol/L ABTS stock solution with 88 μL of 140 mmol/L potassium persulfate solution. The mixture was diluted with absolute ethanol until its absorbance at 734 nm reached 0.70 ± 0.02. For the assay, 0.50 mL of the serum was mixed with 2.00 mL of this ABTS working solution. The reaction was carried out in the dark for 6 min, after which the absorbance was measured at 734 nm.

The scavenging activity against each free radical was calculated using the following formula:

$$\text{Radical scavenging rate}\;\left(\%\right)=\left[\left(A_{0}-\left(A_{1}-A_{2}\right)\right)/A_{0}\right]\times 100\%$$

A_0_ is the absorbance of the blank control; A_1_ is the absorbance after adding the sample; A_2_ is the absorbance of the sample itself.

### 2.5 Determination of Anti‐Lipid Peroxidation (ALP) Activity

2.00 mL of serum, 1.00 mL of 10 mg/mL soybean lecithin solution, and 1.00 mL of 0.4 mmol/L ferrous sulfate solution were added to a test tube. The tube was incubated in a 37°C water bath for 60 min, followed by the addition of 1 mL of TCA‐TBC‐HCl solution. The mixture was then incubated in a 95°C water bath for 15 min. The absorbance was measured at 535 nm.

$$\text{Anti}-\text{lipid peroxidation activity}\;\left(\%\right)=\left[A_{0}-\left(A_{1}-A_{2}\right)\right]/A_{0}\times 100\%$$

A_0_ is the absorbance when water is used instead of the sample solution, A_1_ is the absorbance of sample solution after reaction, A_2_ is the control absorbance when water is used instead of lecithin solution.

### 2.6 Determination of Antioxidant Enzymes and Proinflammatory Factors

The serum activities of catalase and superoxide dismutase (both from Beijing Solarbio Science & Technology Co. Ltd.), as well as the concentrations of interleukin‐6 (IL‐6), interleukin‐1β (IL‐1β), and tumor necrosis factor‐α (TNF‐α) (all from Wuhan Purity Biotechnology Co. Ltd.), were measured according to the instructions provided with the respective assay kits.

### 2.7 Quantitative Real‐Time PCR (qPCR) Assay

Gene‐specific primers for Gpx1 (F‐CAATCAGTTCGGGACATCAGGAG; R‐TCACCATTCACCTCGCACTT) and Gclm (F‐TTAATCTTGCCTCCTGCTGTGT; R‐CACTCCTGGGCTTCAATGTCA) were synthesized by Wuhan Genecreate Biological Engineering Co. Ltd. The qPCR reaction system: 10 μL of 2× Universal Blue SYBR Green qPCR Master Mix, 1.0 μL of 10 μM gene primer, and 1.0 μL of 10 μM reverse transcription product were taken in a 0.2 mL PCR tube, and then Nuclease‐Free Water was added to volume to 20.0 μL. The thermal cycling conditions were as follows: initial denaturation at 95°C for 30 s; followed by 45 cycles of denaturation at 95°C for 15 s and annealing/extension at 60°C for 30 s. Each sample was run in triplicate, and the GAPDH gene was used as an internal control. The relative mRNA expression levels of the target genes were calculated using the 2^(−ΔΔCt) method.

### 2.8 Western Blotting Analysis

A portion of liver tissue stored at −80°C was used for Western blot analysis according to a method of Mao (Mao et al. 2024). Protein was extracted from liver homogenate. Tissue blocks were cut into small pieces and placed in a homogenizing tube, and 10 times the volume of lysate was added to the tissue. The lysate was then placed on ice for 30 min with shaking every 5 min to ensure complete tissue lysis. Then, it was centrifuged at 10,000 rpm, 4°C for 10 min, and the supernatant was placed in a 1.5 mL centrifuge tube and stored at −80°C. Then, the protein content was measured using a BCA protein assay kit (Aidlab Biotechnologies Co. Ltd., Beijing, China). Subsequently, SDS—PAGE electrophoresis, immunoblotting, and chemiluminescence gel imaging were performed, respectively.

### 2.9 Interactive Gut Microbial Community Diversity Analysis

Samples of intestinal contents were collected from rats belonging to the control group (group A), model group (group B), positive control group (group C), and H‐AGF treatment group (group D) to analyze microbial diversity. The obtained PCR products were pooled and separated on a 2% agarose gel. Subsequently, the AxyPrep DNA Gel Extraction Kit (from Axygen Biosciences, Union City, CA, USA) was employed to purify these PCR products, while their quantification was carried out using the Quantus Fluorometer (Promega, USA). The NEXTFLEX Rapid DNA‐Seq Kit was utilized for constructing the sequencing library. Finally, the Illumina Miseq PE300/NovaSeq PE250 platform (provided by Shanghai Majorbio Bio‐pharm Technology Co. Ltd.) was used for sequencing purposes.

## 3 Results and Analysis

### 3.1 Total Antioxidant Capacity, Reducing Power, and Anti‐Lipid Peroxidation Activity Were Determined

**FIGURE 1:**
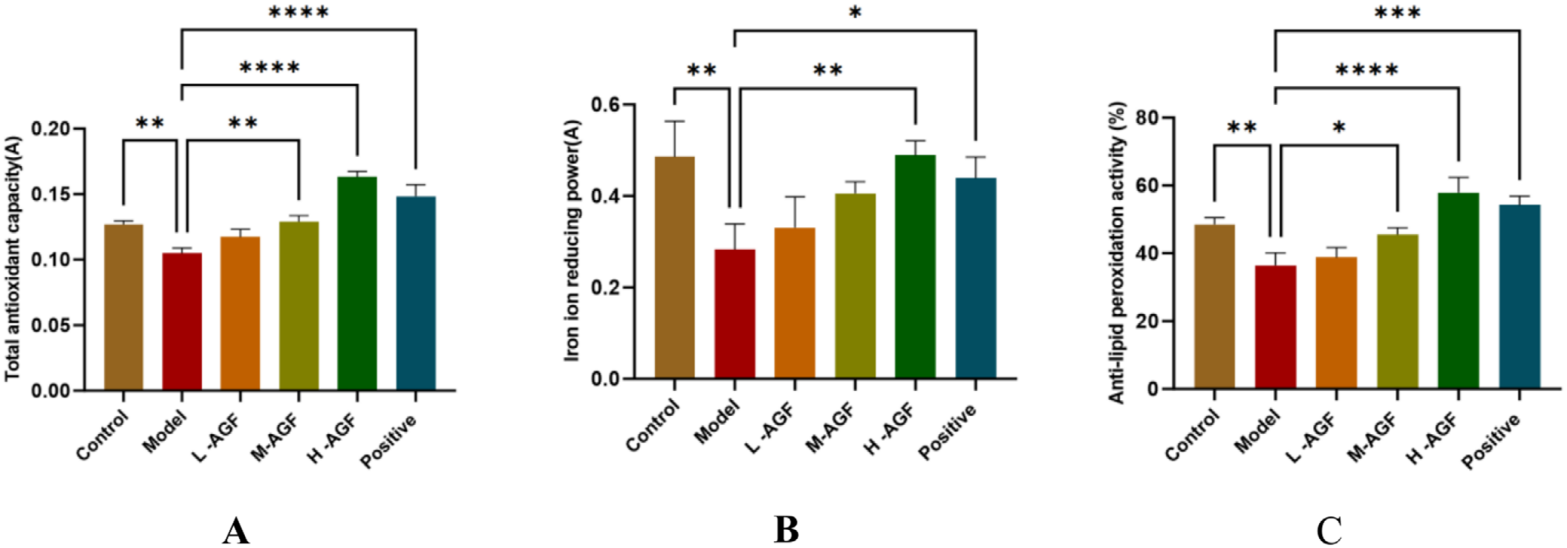
Results of H‐AGF determination of total antioxidant capacity (A), reducing power (B), and anti‐lipid peroxidation activity (C) in rat serum. Asterisks indicate statistical significance: *p < 0.05; **p < 0.01; ***p < 0.001; ****p < 0.0001.

The AGF administration significantly enhanced both the total antioxidant capacity and ferric ion reducing power in rat serum (Figure 1A,B). The high‐fat model group showed values of 0.105A for antioxidant capacity and 0.283A for reducing power, which is similar to previous results (Shi et al. 2026). Following AGF intake, both parameters increased in a dose‐dependent manner with rising flavonoid levels, reaching 0.163A and 0.489A, respectively, in the high‐flavonoid high‐fat group—exceeding the control group values of 0.127A and 0.486A.

Likewise, the AGF can enhance the anti‐lipid peroxidation activity in rat serum (Figure 1C). The anti‐lipid peroxidation activity in the high‐fat model group was 36.46%. After the administration of AGF flavonoids, there was a marked increase in the serum anti‐lipid peroxidation activity of rats, and as the flavonoid dosage increased, the anti‐lipid peroxidation activity became stronger, reaching 57.89% in the high‐flavonoid high‐fat group, which was higher than that in the control group (48.47%).

### 3.2 Free Radical Scavenging Capacity (FRSC) Assay Results

**FIGURE 2:**
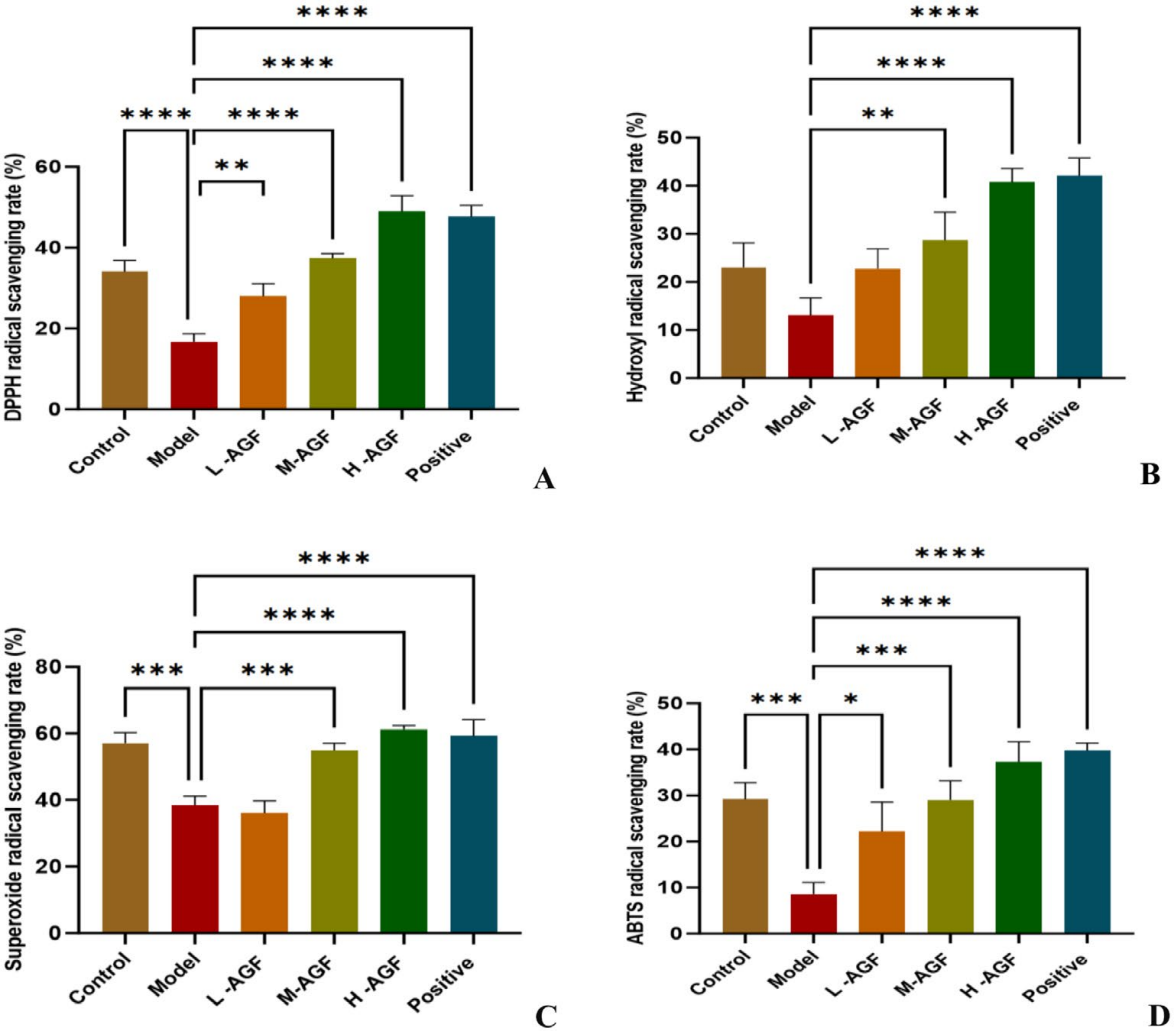
The results of H‐AGF scavenging rate of DPPH free radicals (A), hydroxyl free radicals (B), superoxide anion free radicals (C), and ABTS free radicals (D) in rat serum. Asterisks indicate statistical significance: *p < 0.05; **p < 0.01; ***p < 0.001; ****p < 0.0001.

The study demonstrated that AGF significantly enhanced free radical scavenging capacity in rat serum. As shown in Figure 2A, the DPPH radical scavenging rate was only 16.67% in the hyperlipidemic model group, but increased dose‐dependently with AGF supplementation, reaching 48.99% in the high‐dose flavonoid group, which was higher than both the normal control (34.09%) and Xuezhikang control (47.73%) groups.

Similarly, hydroxyl radical scavenging capacity (Figure 2B) improved from 13.08% in the model group to 40.75% in the high‐dose group, surpassing the normal control (23.02%), though slightly lower than the Xuezhikang group (42.14%). For superoxide anion radicals (Figure 2C), the scavenging rate increased from 38.42% to 61.26% with high‐dose treatment, exceeding both control groups (normal: 57.04%; Xuezhikang: 59.17%). The ABTS radical scavenging assay (Figure 2D) showed comparable results, with rates improving from 8.58% to 37.33% in the high‐dose group, again higher than normal controls (29.21%) but marginally below Xuezhikang (39.78%). These findings indicate that the intervention dosage of flavonoids from AGF significantly enhances the free radical scavenging capacity in rat serum, and the effects in the high‐dose group are generally stronger than those in the XZK‐treatment group.

### 3.3 Results of Antioxidant Enzyme Activity Assays

**FIGURE 3:**
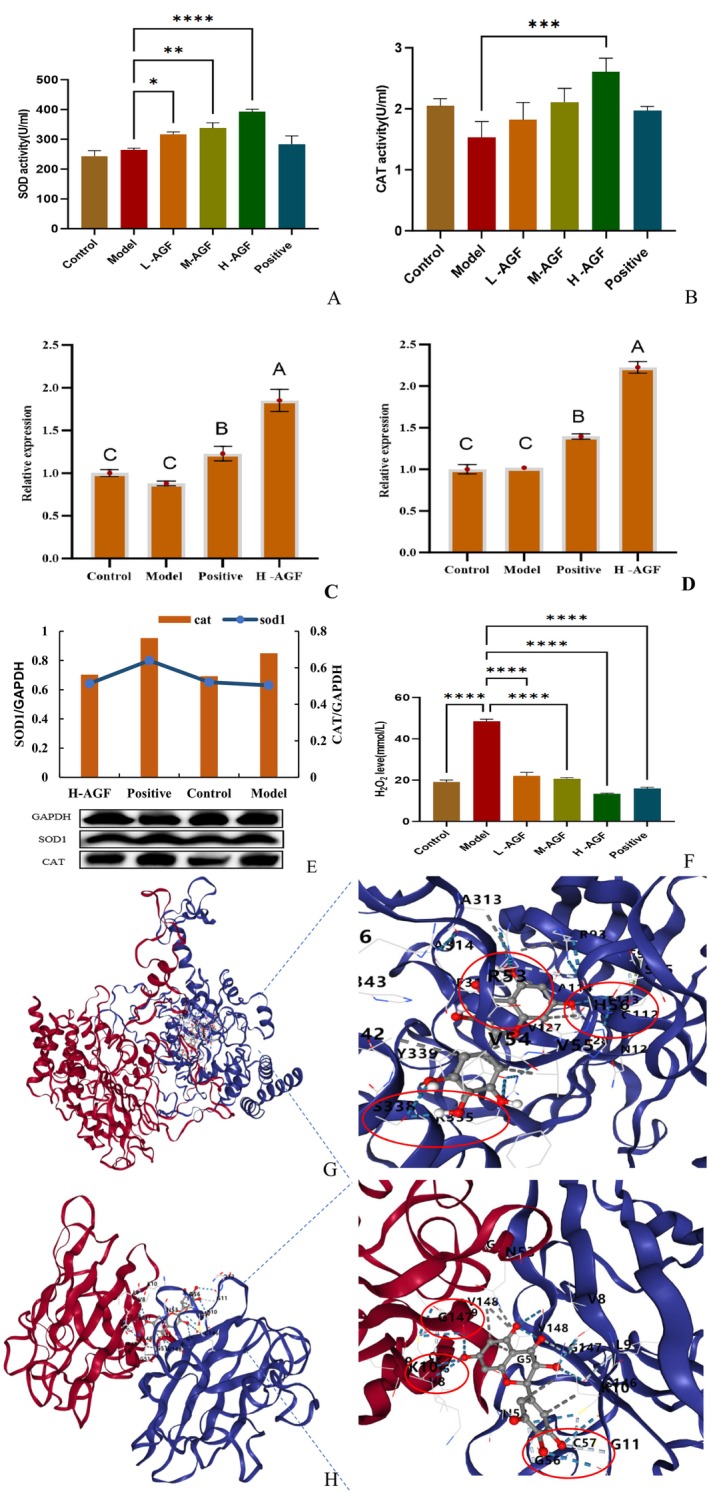
Effects of H‐AGF on SOD (A) and CAT (B) activities, sod1 (C) and CAT (D) gene expression levels, related genes (E), hydrogen peroxide content (F) in rat serum, and molecular docking diagrams of dihydromyricetin with CAT (G) and SOD (H). Asterisks indicate statistical significance: *p < 0.05; **p < 0.01; ***p < 0.001; ****p < 0.0001.

The AGF significantly enhanced serum SOD (Figure 3A) and CAT (Figure 3B) activities in rats. The hyperlipidemic model group showed baseline enzyme activities of 264.96 U/mL (SOD) and 1.536 U/mL (CAT). A dose‐dependent increase in antioxidant enzyme activities was observed with flavonoid supplementation, reaching 392.84 U/mL (SOD) and 2.605 U/mL (CAT) in the high‐dose flavonoid group—surpassing both normal control and XZK‐treated groups. At the genetic level, the AGF upregulated hepatic SOD1 (Figure 3C) and CAT (Figure 3D) gene expression. Compared to the model group, the high‐dose flavonoid group exhibited 1.852‐fold (SOD1) and 2.225‐fold (CAT) increases in gene expression, exceeding both control groups. Protein expression analysis (Figure 3E) revealed SOD1 (0.642 vs. 0.630) and CAT (0.562 vs. 0.554) levels in the high‐dose group were slightly elevated compared to the model group, though marginally lower than XZK‐group values. Notably, the AGF effectively reduced serum hydrogen peroxide levels (Figure 3F) from 48.50 mmol/L in model group to 13.36 mmol/L in the high‐dose group, demonstrating superior efficacy to both control groups.

For further analysis, dihydromyricetin was selected for visualization (Figure 3G,H). Molecular docking results revealed that dihydromyricetin formed hydrogen bonds with the respective target proteins (with a binding energy of—9.7 kcal·mol^−1^ for CAT and—8.1 kcal·mol^−1^ for SOD). Specifically, dihydromyricetin established two hydrogen bonds with residue S338 and one hydrogen bond each with residues R53 and H56 on CAT. Similarly, it formed three hydrogen bonds with residues V8, K10, and G147, and one hydrogen bond with residues G56 and C57 on SOD. Additionally, the presence of π‐π or π‐σ interactions was observed. These molecular docking results suggest that dihydromyricetin likely plays a significant role in alleviating high‐fat‐induced oxidative stress by effectively binding to these key antioxidant enzymes.

### 3.4 Effects of AGF on Pro‐Inflammatory Factors in Rats

**FIGURE 4:**
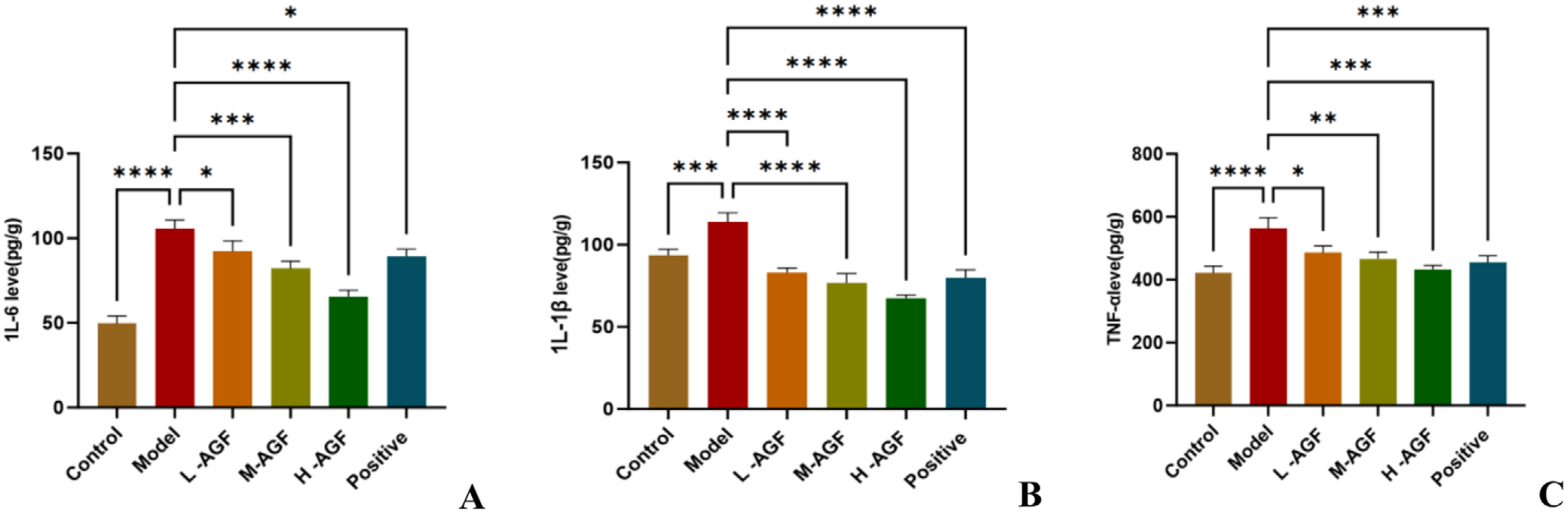
Effects of AGF on liver pro‐inflammatory factors 1 L‐6 (A), 1 L‐1β (B), and TNF‐α (C) levels in rats. Asterisks indicate statistical significance: *p < 0.05; **p < 0.01; ***p < 0.001; ****p < 0.0001.

The control group exhibited low serum levels of pro‐inflammatory factors, while the model group showed the highest concentrations. Administration of different doses of AGF and Xuezhikang significantly reduced serum pro‐inflammatory factor levels (Figure 4). However, the anti‐inflammatory efficacy of Xuezhikang was inferior to that of the H‐AGF group. In the H‐AGF group, levels of IL‐6, IL‐1β, and TNF‐α were decreased by 37.93%, 40.65%, and 23.16% respectively, compared to the model group. Hepatic concentrations of these pro‐inflammatory factors (IL‐6, IL‐1β, and TNF‐α) demonstrated a dose‐dependent decrease with increasing AGF supplementation. These results indicate that AGF can effectively reduce the levels of pro‐inflammatory factor (IL‐6, IL‐1β, TNF‐α) in a dose‐dependent manner, thereby mitigating inflammatory damage.

### 3.5 Effects of Flavonoid‐Mediated Intestinal Microbiota on Blood Lipids in Rats

#### 3.5.1 Alphadiversity Analysis

As shown in Table 2, the microbial richness indices (Chao, ACE, Sobs) and diversity indices (Shannon, Simpson) were the highest in the basal diet group (Group A). These metrics decreased in the high‐fat diet group (Group B) compared to Group A. Compared to Group B, the richness and diversity were slightly lower in the high‐fat diet + flavonoid group (Group D), while a more significant reduction was observed in the high‐fat diet + Xuezhikang group (Group C). These findings indicate that a high‐fat diet decreases microbial richness and diversity and that flavonoids have a superior mitigating effect on this reduction compared with Xuezhikang.

**TABLE 2:** Microbial diversity index table.

Sample	Abundance			Diversity	
	Chao	Ace	Sobs	Simpson	Shannon
Control group (A)	232.83	233.72	211.33	0.0867	3.0167
Model group (B)	217.19	222.91	193.67	0.1767	2.5000
Xuezhikang group (C)	183.4	181.23	157.33	0.22	2.1000
H‐AGF group (D)	211.44A	220.44	192.33	0.2067	2.3667

*Note:* the first column in the table is the sample name, and the subsequent columns respectively correspond to the calculated results of Chao1, ACE, Shannon, Simpson, and other diversity indexes of each sample at the same sequencing depth. Different capital letters in the table indicate the extremely significant level of each index data.

#### 3.5.2 Coverage Curve and Venn Analysis

**FIGURE 5:**
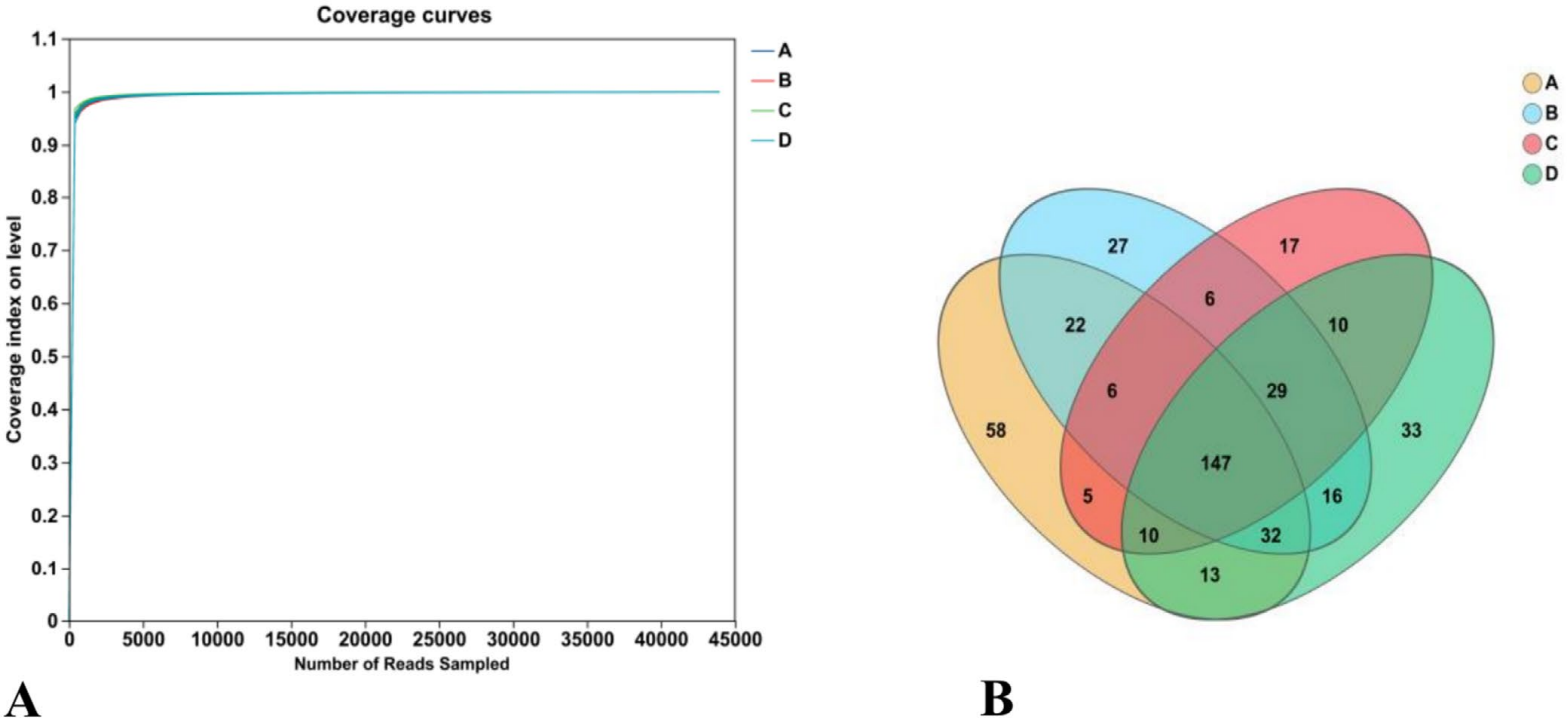
(A) OTU dilution curve analysis; (B) Venn analysis.

The cecal contents of mice were analyzed using 16S rRNA sequencing technology. The α‐diversity analysis based on the coverage curves of each sample reached a saturation plateau (Figure 5A), indicating high reliability of subsequent experiments. The Venn diagram revealed a total of 140 operational taxonomic units (OTUs), with 60 unique to Group A, 27 to Group B, 17 to Group C, and 33 to Group D. Excluding Group A, Group D exhibited the highest number of unique OTUs, suggesting that flavonoids possess a certain ability to modulate microbial structure. The presence of 14 shared OTUs between Groups A and D indicates a degree of similarity between these two groups.

#### 3.5.3 Community Analysis at the Order and Genus Levels

**FIGURE 6:**
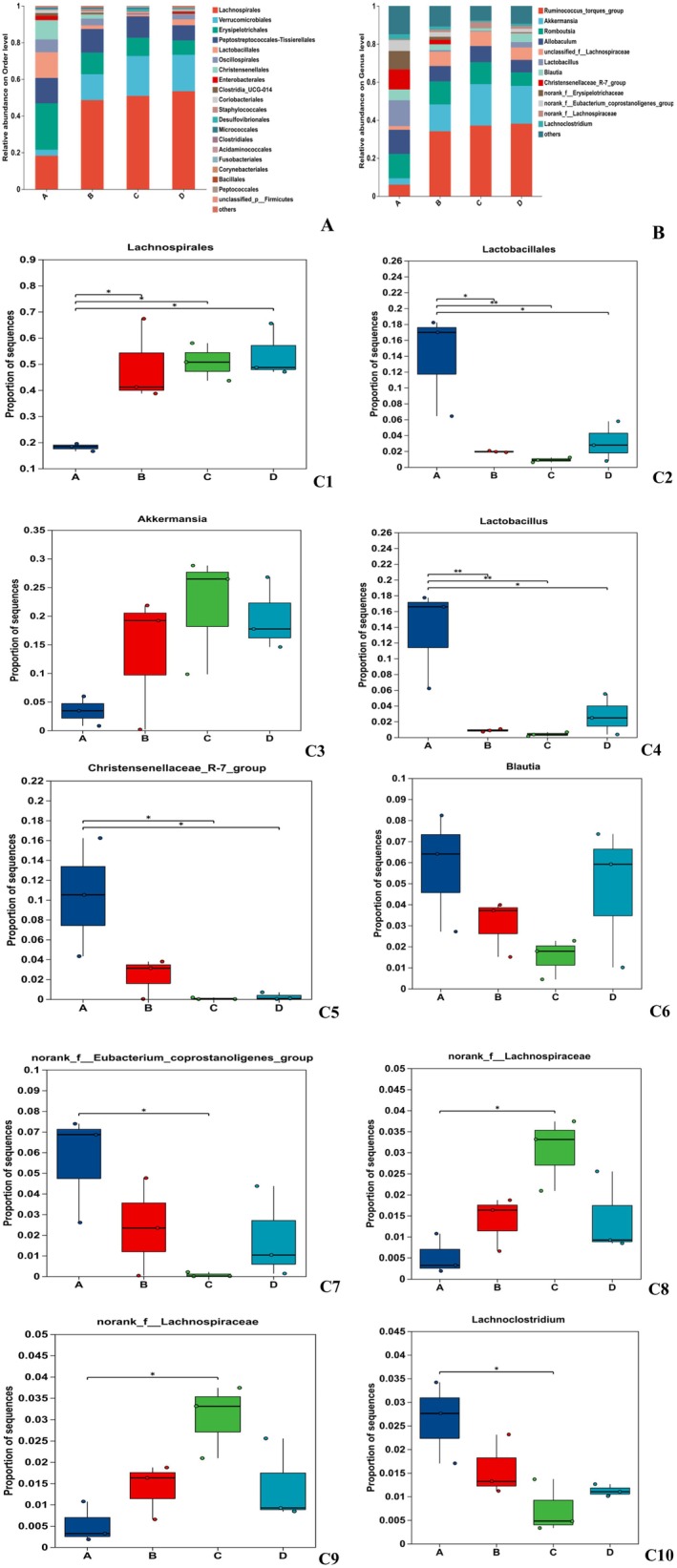
Group distribution of the community at the order (A) and genus (B) levels, along with intergroup differences (C1–10).

To evaluate the overall composition and structure of gut microbiota across different experimental groups, we analyzed the distribution and abundance of bacterial taxa at both the order and genus levels. The *Lachnospirales* showed the highest relative abundance in Group D (Figure 6A). *Lachnospirales* facilitates the production of short‐chain fatty acids (acetate and butyrate) that supply energy to colonic epithelial cells, modulate intestinal pH, and suppress pathogen growth (Kim et al. 2025). Flavonoids (Group D) increased the abundance of *Lactobacillales* (Figure 6A), which improve the effects of prednisone on autoimmune hepatitis via gut microbiota‐mediated follicular helper T cells (Ma et al. 2022). Additionally, Liu showed the negative relationship between Lactobacillales and TC and MDA levels (Liu et al. 2021). Concurrently, flavonoids (Group D) increased the abundance of beneficial microbes such as *Akkermansia*, L*actobacillus*, *Christensenellaceae_R‐7_group*, *Blautia*, and *norank_f__Eubacterium_coprostanoligenes_group*, while reducing the abundance of harmful microbes including *norank_f__Erysipelotrichaceae* and *Lachnoclostridium* (Figure 6B).

#### 3.5.4 Analysis of Differences Between Intestinal Microbiomes

**FIGURE 7:**
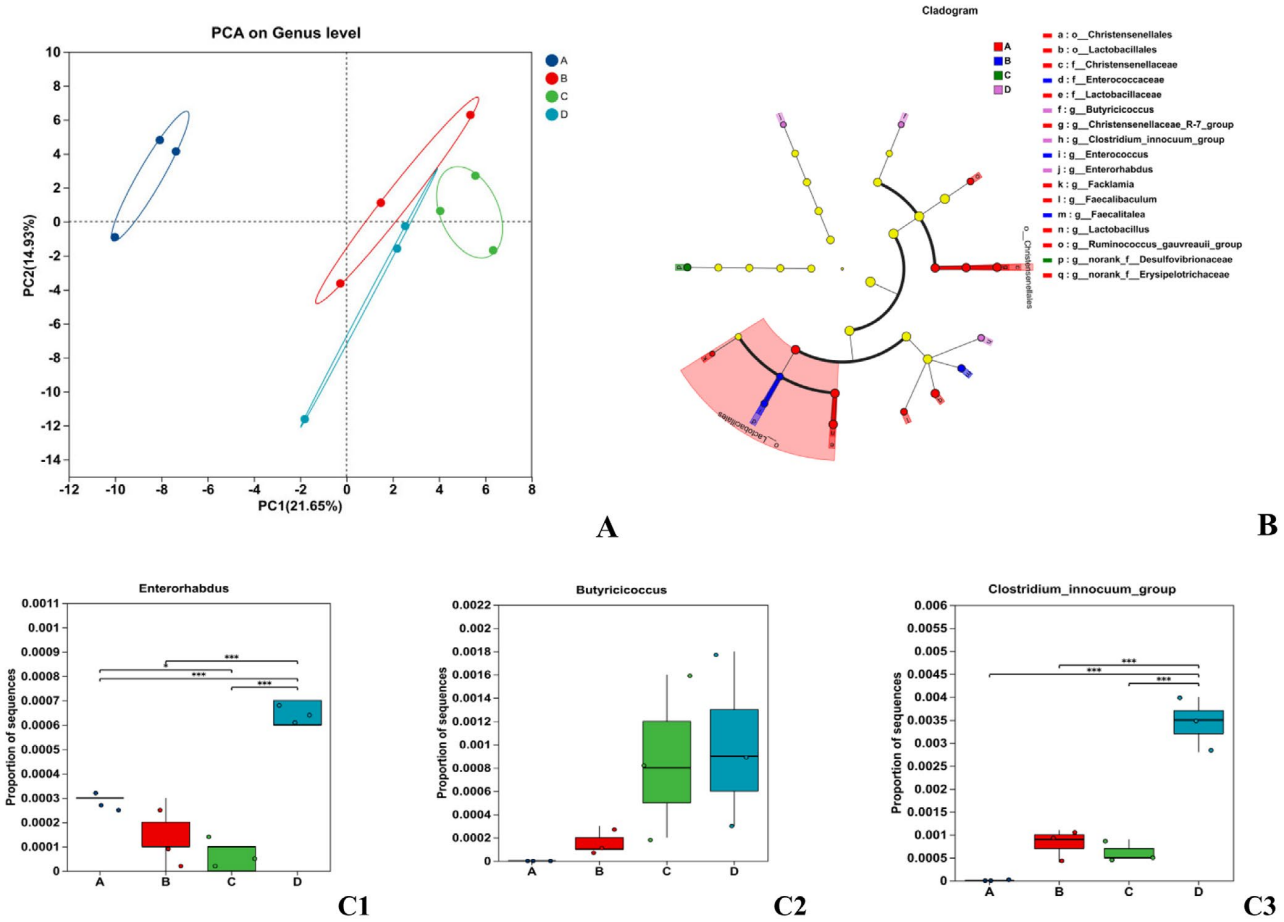
Genus‐level PCA (A), Cladogram (B), and intergroup differences (C1–C3) of gut microbiota in each rat group.

The PCA results showed that principal component 1 separated groups A from B, C, and D (Figure 7A), indicating that the intestinal microbiota of rats fed the basal diet differed from those fed high‐fat diets. Although groups B, C, and D were clustered together, the D group showed significant differences from B and C, indicating that the intestinal microbiota of rats fed flavonoid compounds and a high‐fat diet differed from the other groups (Figure 7A). The distinct microbial taxa identified in Group D were *Enterorhabdus*, *Butyricicoccus*, and *Clostridium_innocuum_group*. *Butyricicoccus* demonstrated significant associations with the *Ruminococcus gauvreauii group* and *Christensenellaceae*, whereas *Clostridium_innocuum_group* exhibited correlations with *Lactobacillales* and *Faecalibaculum* (Figure 7B). These microorganisms predominantly function in short‐chain fatty acid (SCFA) metabolism, regulating the microbial ecosystem equilibrium to sustain intestinal health, as well as exhibiting anti‐inflammatory properties, immune–enhancement capabilities, and obesity–alleviating effects.

#### 3.5.5 Correlation of Intestinal Microflora With Biochemical Indexes

**FIGURE 8:**
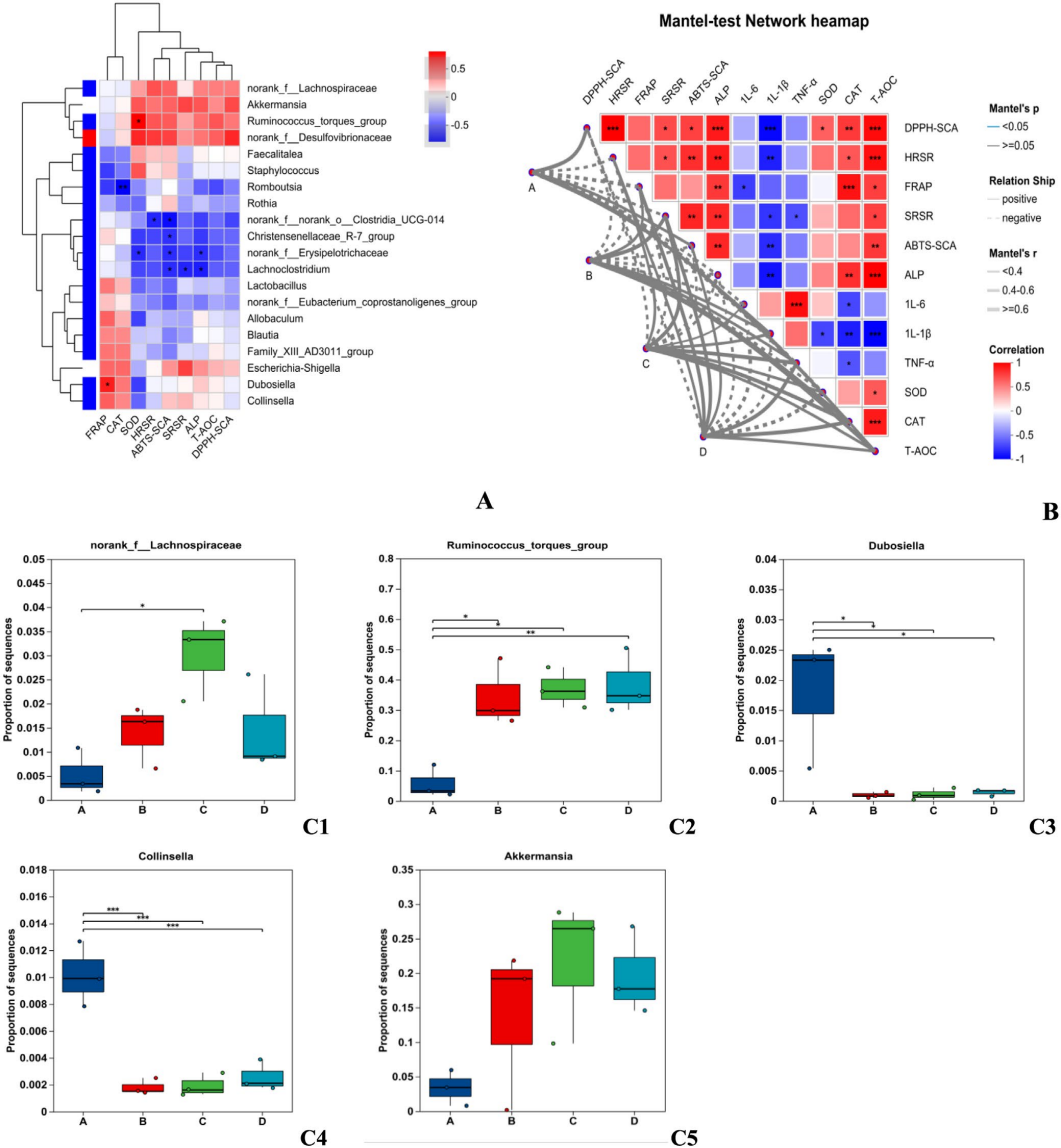
Heatmap of correlation coefficients between gut microbiota and physiological indices (A), network heatmap of Mantel test results for gut microbiota and biochemical indices (B), and intergroup differences (C1–C5). *p < 0.05, **p < 0.01, ***p < 0.001: Significance of Spearman correlation.

The correlation analysis of intestinal microflora with biochemical indexes was investigated using Spearman's correlation coefficient (Figure 8A). The heatmap showed that the *norank_f__Lachnospiraceae, Akkermansia*, and *Ruminococcus_torques_group* were positively correlated with most of the physiological indicators, except for the FRAP. Moreover, *Dubosiella* and *Collinsella* were positively correlated with the FRAP, CAT, ABTS‐SCA, SRSR, ALP, and T‐AOC.

*Dubosiella* and *Collinsella* can enhance immune function. *Ruminococcus_torques_group*, *Dubosiella*, and *Collinsella* promote the growth of beneficial bacteria, enhance immune function, regulate lipid metabolism, and reduce the effects of obesity. Additionally, these microorganisms are more abundant in Group D (Figure 8C).

The Mantel‐test Network heatmap (Figure 8B) heatmap shows that pro‐inflammatory factors (IL‐6, IL‐1β, TNF‐α) were negatively correlated with most of the physiological indicators, except for the SOD. Furthermore, the SOD and CAT were positively correlated with the FRAP, HRSR, ABTS‐SCA, SRSR, ALP, T‐AOC, and DPPH‐SCA. Microbial communities in the H‐AGF (D) group were positively correlated with FRAP, HRSR, ABTS‐SCA, SRSR, ALP, T‐AOC, DPPH‐SCA, and CAT. In particular, the DPPH‐SCA and ALP were only positively correlated with H‐AGF (D). Collectively, the H‐AGF (D) can improve the dysbiosis of intestinal flora induced by hyperlipidemia, thus preventing the occurrence of hyperlipidemia and liver injury due to oxidative stress.

## 4 Conclusion

### 4.1 Antioxidant Capacity of AGF

Alcohol consumption induces oxidative stress, which is one of the primary causes leading to the production of oxygen‐free radicals in the liver, triggering inflammation and cell death. Numerous studies have demonstrated that natural flavonoids play significant roles in scavenging free radicals, enhancing antioxidant activity, and inhibiting cancer cell proliferation (Tumilaar et al. 2024; Ahmad and Ghosh 2022). Our study found that rats in the AGF‐H group exhibited higher serum antioxidant enzyme (SOD, CAT) activity, along with significantly enhanced antioxidant capacity, free radical scavenging ability, and reduced membrane lipid peroxidation (Figures 1 and 3).

Moreover, this finding aligns with observations reported by Guo, as well as Molina and Boadi. Guo found that dietary supplementation with dihydromyricetin in pigs increased the levels of serum total superoxide dismutase (T‐SOD), reduced glutathione (GSH) in both serum and liver, muscle catalase (CAT), and serum high‐density lipoprotein cholesterol (HDL‐C), while decreasing hepatic malondialdehyde (MDA) and muscle triglyceride (TG) levels (Guo et al. 2021). The increase in the enzymatic activities of SOD and CAT may be related to the effective binding of dihydromyricetin to SOD and CAT proteins (Figure 3G,H). Molina demonstrated that quercetin supplementation in mice with ethanol‐induced oxidative stress enhanced the activities of superoxide dismutase (SOD), catalase (CAT), glutathione peroxidase (GPx), glutathione reductase (GR), and glutathione (GSH), while significantly reducing malondialdehyde (MDA) levels (Molina et al. 2003). Boadi demonstrated that flavonoids reduced lipid peroxide levels and increased glutathione levels in mixed human liver microsomes (Boadi et al. 2021). Furthermore, our study revealed that AGF decreased pro‐inflammatory factor levels (Figure 4). Similarly, Zhao found that dihydromyricetin significantly ameliorated liver fibrosis in carbon tetrachloride (CCl₄)‐induced model mice, concurrently elevating superoxide dismutase (SOD) and glutathione (GSH) levels while reducing malondialdehyde (MDA), IL‐6, IL‐1β, and TNF‐α levels (Zhao et al. 2021).

### 4.2 Regulating Gut Microbiota Capacity of PVE

Flavonoids can modulate microbial composition by optimizing the structure of microbial communities, thereby creating an internal environment conducive to host health. This process enhances the body's antioxidant defenses, strengthens immune system function, and improves overall immunity (Chagas et al. 2022). In our study, the flavonoid group increased the abundance of beneficial microbes, including *Akkermansia*, *Lactobacillus*, *Christensenellaceae*_R‐7_group, *Blautia*, *norank_f__Eubacterium_coprostanoligenes*.

*_group* (Figure 6B), *Enterorhabdus*, *Butyricicoccus*, and *Clostridium_innocuum_group* (Figure 7B), as well as *norank_f__Lachnospiraceae*, *Akkermansia*, and *Ruminococcus_torques_group*, *Dubosiella* and *Collinsella* (Figure 8C). Moreover, these findings are consistent with observations by Zhang, as well as Cheng, Wu, and Liu. Cheng found that chitosan oligosaccharides (COS) may regulate immune function and antioxidant activity by enhancing probiotics such as *Lactobacillus*, *Collinsella*, *Blautia*, and *Lachnospiraceae*_NK4A136_group (Cheng et al. 2024). Wu demonstrated that *Akkermansia muciniphila* enhanced the diversity and richness of the gut microbiota in mice, modulated the abundance of specific microbial taxa, including *Lachnospiraceae* and *Ruminococcaceae*, improved hepatic immune capacity, and significantly attenuated hepatocyte apoptosis (Wu et al. 2017). Zhang (Zhang, Tu, et al. 2024) and Liu (Liu et al. 2023) demonstrated that *Dubosiella newyorkensis* exerts immunomodulatory probiotic effects on DSS‐induced colitis, concurrently reducing malondialdehyde (MDA) levels and increasing superoxide dismutase (SOD) activity in mice. These changes alleviated oxidative stress, improved vascular endothelial function, and modulated gut microbiota composition. Zhang found that *Ruminococcus torques* significantly ameliorated inflammation, liver fibrosis, and fatty liver symptoms in mice induced by a high‐fat diet, with a more than 40% reduction in the expression of TNF‐α (Zhang, Wang, et al. 2024). Furthermore, our study revealed that microorganisms such as *norank_f__Lachnospiraceae*, *Akkermansia*, *Ruminococcus_torques_group*, *Dubosiella*, and *Collinsella* exhibited positive correlations with most antioxidant physiological indicators. This further demonstrates that AGF can enhance the body's antioxidant capacity by modulating microbial composition. However, the precise mechanisms through which vine tea flavonoids enhance serum immune protein levels, thereby boosting systemic immunity and antioxidant capacity in high‐fat‐fed rats by modulating gut microbial architecture, require further investigation.

In addition, flavonoids can optimize microbial community structure to potentiate the liver's lipid‐lowering functions (Zhou et al. 2024). Zeng demonstrated that *Butyricicoccus*, *Bifidobacterium*, and *Enterorhabdus*, etc., were cholesterol‐lowering probiotics and they decreased cholesterol levels by promoting the conversion of cholesterol into BAs and then promoting the excretion of BAs in fecal matter (Zeng et al. 2024). Xu revealed that *Akkermansia*, *Clostridium_IV*, *Lactobacillus*, *Butyricicoccus*, and others were negatively correlated with serum levels of TC, TG, LDL‐C, ALT, and HOMA‐IR, but positively correlated with HDL‐C and fecal TBA levels (Xu et al. 2022). Future research should integrate multi‐omics technologies to comprehensively elucidate the hepatoprotective targets of flavonoids and their cascade regulatory mechanisms, while advancing preclinical translation to establish a scientific foundation for developing functional liver‐protective foods and pharmaceuticals.

## 5 Conclusion

In summary, this study demonstrates that H‐AGF significantly enhanced both protein levels and gene expression of SOD and CAT in hyperlipidemic rats. Moreover, H‐AGF administration elevated SOD and CAT enzyme activities, total antioxidant capacity, reducing power, and anti‐lipid peroxidation capability in rats. These improvements consequently increased the scavenging rates of DPPH, hydroxyl, superoxide anion, and ABTS free radicals, effectively mitigating hepatic oxidative damage induced by hyperlipidemia in rats.

Simultaneously, H‐AGF treatment significantly reduced levels of pro‐inflammatory cytokines (IL‐6, IL‐1β, and TNF‐α) in rats. Additionally, H‐AGF ameliorated gut microbiota dysbiosis induced by hyperlipidemia. Notably, several microbial taxa including *Enterorhabdus*, *Butyricicoccus*, *Clostridium_innocuum_group*, *norank_f__Lachnospiraceae*, *Akkermansia*, *Ruminococcus_torques_group*, *Dubosiella*, and *Collinsella* were found to be closely associated with enhanced antioxidant capacity, improved immune function, regulated lipid metabolism, and maintained liver health.

This study provides an innovative strategy for modulating oxidative damage, highlighting the substantial potential of H‐AGF in developing functional foods and pharmaceuticals with antioxidant properties.

## Author Contributions

**Li Lan Lei:** methodology, writing – original draft, software, data curation. **Hao Shi:** conceptualization, methodology, software, data curation, formal analysis, validation, investigation, funding acquisition, writing – original draft, visualization, writing – review and editing, project administration, resources, supervision. **Jing Wang:** software, formal analysis, project administration, resources. **Bo Zhou:** methodology, writing – original draft, software, data curation.

## Funding

This work was supported by Natural Science Foundation of Hunan Province, China (2023JJ40468). Hunan Provincial Engineering Technology Research Centre for Fresh Wet Rice Noodle Processing (2025KF12). Changde Engineering Research Centre forEcological Process Regulation and High‐value Utilisation of Economic Forests; Hunan “14 th Five‐Year Plan” Applied Characteristic Disciplines (Forestry) (Xiang jiao tong (2022) 351).

## Data Availability

The data that support the findings of this study are available from the corresponding author upon reasonable request.

## References

[fsn371746-bib-0001] Ahmad, S. R. , and P. Ghosh . 2022. “A Systematic Investigation on Flavonoids, Catechin, β‐Sitosterol and Lignin Glycosides From Saraca Asoca (Ashoka) Having Anti‐Cancer & Antioxidant Properties With no Side Effect.” Journal of the Indian Chemical Society 99, no. 1: 100293.

[fsn371746-bib-0002] Boadi, W. Y. , C. Stevenson , D. Johnson , and M. A. Mohamed . 2021. “Flavonoids Reduce Lipid Peroxides and Increase Glutathione Levels in Pooled Human Liver Microsomes (HLMs).” Advances in Biological Chemistry 11, no. 6: 283–295. 36340955 10.4236/abc.2021.116019 PMC9634994

[fsn371746-bib-0003] Carneiro, R. C. , L. Ye , N. Baek , G. H. Teixeira , and S. F. O'Keefe . 2021. “Vine Tea (Ampelopsis Grossedentata): A Review of Chemical Composition, Functional Properties, and Potential Food Applications.” Journal of Functional Foods 76: 104317.

[fsn371746-bib-0004] Chagas, M. S. S. , M. D. Behrens , C. J. Moragas‐Tellis , G. X. Penedo , A. R. Silva , and C. F. Gonçalves‐de‐Albuquerque . 2022. “Flavonols and Flavones as Potential Anti‐Inflammatory, Antioxidant, and Antibacterial Compounds.” Oxidative Medicine and Cellular Longevity 2022, no. 1: 9966750. 36111166 10.1155/2022/9966750 PMC9470311

[fsn371746-bib-0005] Cheng, G. , T. Hu , Y. Zeng , et al. 2024. “Enhancing Immune Response, Antioxidant Capacity, and Gut Health in Growing Beagles Through a Chitooligosaccharide Diet.” Frontiers in Veterinary Science 10: 1283248. 38274661 10.3389/fvets.2023.1283248 PMC10808298

[fsn371746-bib-0006] Gao, Y. , H. Wu , Y. Luo , X. Deng , J. Chen , and T. Wu . 2025. “Mechanisms of Dihydromyricetin for Improving Hepatic Fibrosis Through the Integration of Metabolomics and Gut Microbiota.” American Journal of Chinese Medicine 53, no. 3: 889–908. 40374379 10.1142/S0192415X25500338

[fsn371746-bib-0007] Guo, Z. , X. Chen , Z. Huang , et al. 2021. “Dietary Dihydromyricetin Supplementation Enhances Antioxidant Capacity and Improves Lipid Metabolism in Finishing Pigs.” Food & Function 12, no. 15: 6925–6935. 34132271 10.1039/d0fo03094e

[fsn371746-bib-0008] Kim, Y.‐I. , W. Choi , M. Seo , S. Ka , and J. Park . 2025. “Effect of Exercise on the Human Gut Microbiota in Individuals With Overweight and Obesity: A Systematic Review and Meta‐Analysis of Randomized Controlled Trials.” Physical Activity and Nutrition 29, no. 2: 49. 40765072 10.20463/pan.2025.0014 PMC12325879

[fsn371746-bib-0009] Liu, H. , H. Zhu , H. Xia , et al. 2021. “Different Effects of High‐Fat Diets Rich in Different Oils on Lipids Metabolism, Oxidative Stress and Gut Microbiota.” Food Research International 141: 110078. 33641963 10.1016/j.foodres.2020.110078

[fsn371746-bib-0010] Liu, T.‐h. , J. Wang , C.‐y. Zhang , et al. 2023. “Gut Microbial Characteristical Comparison Reveals Potential Anti‐Aging Function of Dubosiella Newyorkensis in Mice.” Frontiers in Endocrinology 14: 1133167. 36798665 10.3389/fendo.2023.1133167 PMC9928160

[fsn371746-bib-0011] Lv, H. , T. Xv , J. Peng , et al. 2022. “Dihydromyricetin Improves Liver Fat Deposition in High‐Fat Diet‐Induced Obese Mice.” Journal of Chinese Pharmaceutical Sciences 31, no. 11: 824–838.

[fsn371746-bib-0012] Lyu, Q. , L. Chen , S. Lin , H. Cao , and H. Teng . 2022. “A Designed Self‐Microemulsion Delivery System for Dihydromyricetin and Its Dietary Intervention Effect on High‐Fat‐Diet Fed Mice.” Food Chemistry 390: 132954. 35551031 10.1016/j.foodchem.2022.132954

[fsn371746-bib-0013] Lyu, Q. , H. Deng , S. Wang , et al. 2023. “Dietary Supplementation With Casein/Cyanidin‐3‐O‐Glucoside Nanoparticles Alters the Gut Microbiota in High‐Fat Fed C57BL/6 Mice.” Food Chemistry 412: 135494. 36736183 10.1016/j.foodchem.2023.135494

[fsn371746-bib-0014] Ma, L. , L. Zhang , Y. Zhuang , Y. Ding , and J. Chen . 2022. “Lactobacillus Improves the Effects of Prednisone on Autoimmune Hepatitis via Gut Microbiota‐Mediated Follicular Helper T Cells.” Cell Communication and Signaling 20, no. 1: 83. 35658901 10.1186/s12964-021-00819-7 PMC9166466

[fsn371746-bib-0015] Mao, S.‐Y. , S.‐K. Suo , Y.‐M. Wang , C.‐F. Chi , and B. Wang . 2024. “Systematical Investigation on Anti‐Fatigue Function and Underlying Mechanism of High Fischer Ratio Oligopeptides From Antarctic Krill on Exercise‐Induced Fatigue in Mice.” Marine Drugs 22, no. 7: 322. 39057431 10.3390/md22070322 PMC11278274

[fsn371746-bib-0016] Molina, M. F. , I. Sanchez‐Reus , I. Iglesias , and J. Benedi . 2003. “Quercetin, a Flavonoid Antioxidant, Prevents and Protects Against Ethanol‐Induced Oxidative Stress in Mouse Liver.” Biological and Pharmaceutical Bulletin 26, no. 10: 1398–1402. 14519943 10.1248/bpb.26.1398

[fsn371746-bib-0017] Shi, H. , M.‐l. Qin , Q.‐y. Liu , et al. 2025. “The Vine Tea Flavonoids Extraction by Ultrasound‐Enzyme‐Assisted and Its Consequences for Antioxidant Efficacy and Gut Microbiota in Rats.” Food and Agricultural Immunology 36, no. 1: 2470131.

[fsn371746-bib-0018] Shi, H. , T. Yang , D. D. Tang , and J. Wang . 2026. “Vine Tea Flavonoids Alleviate High‐Fat Diet‐Induced Hyperlipidaemia in Rats.” Food and Agricultural Immunology 37, no. 1: 2608131.

[fsn371746-bib-0019] Teng, F. , and H. Wang . 2025. “Protective Effects and Metabolomics Analysis of Dihydromyricetin on Cyclophosphamide‐Induced Hepatotoxicity in Mice.” Pharmaceutical Science Advances 3: 100063. 41550664 10.1016/j.pscia.2025.100063 PMC12709985

[fsn371746-bib-0020] Tumilaar, S. G. , A. Hardianto , H. Dohi , and D. Kurnia . 2024. “A Comprehensive Review of Free Radicals, Oxidative Stress, and Antioxidants: Overview, Clinical Applications, Global Perspectives, Future Directions, and Mechanisms of Antioxidant Activity of Flavonoid Compounds.” Journal of Chemistry 2024, no. 1: 5594386.

[fsn371746-bib-0021] Vilela, D. L. , P. G. Fonseca , S. L. Pinto , and J. Bressan . 2021. “Influence of Dietary Patterns on the Metabolically Healthy Obesity Phenotype: A Systematic Review.” Nutrition, Metabolism, and Cardiovascular Diseases 31, no. 10: 2779–2791. 10.1016/j.numecd.2021.05.007 34340900

[fsn371746-bib-0022] Wang, L. , H. Zhang , M. Xie , et al. 2024. “Optimization of Brewing Process Vine Tea and Flavor Analysis of Different Brewing Processes.” Journal of Food Biochemistry 2024, no. 1: 8858457.

[fsn371746-bib-0023] Wu, R.‐R. , X. Li , Y.‐H. Cao , et al. 2023. “China Medicinal Plants of the Ampelopsis Grossedentata—A Review of Their Botanical Characteristics, Use, Phytochemistry, Active Pharmacological Components, and Toxicology.” Molecules 28, no. 20: 7145. 37894624 10.3390/molecules28207145 PMC10609530

[fsn371746-bib-0024] Wu, W. , L. Lv , D. Shi , et al. 2017. “Protective Effect of *Akkermansia muciniphila* Against Immune‐Mediated Liver Injury in a Mouse Model.” Frontiers in Microbiology 8: 1804. 29033903 10.3389/fmicb.2017.01804 PMC5626943

[fsn371746-bib-0025] Xu, W. , K. Zou , Y. Zhan , et al. 2022. “ *Enterococcus faecium* GEFA01 Alleviates Hypercholesterolemia by Promoting Reverse Cholesterol Transportation via Modulating the Gut Microbiota‐SCFA Axis.” Frontiers in Nutrition 9: 1020734. 36424921 10.3389/fnut.2022.1020734 PMC9678928

[fsn371746-bib-0026] Zeng, T. , Y. Song , S. Qi , R. Zhang , L. Xu , and P. Xiao . 2023. “A Comprehensive Review of Vine Tea: Origin, Research on Materia Medica, Phytochemistry and Pharmacology.” Journal of Ethnopharmacology 317: 116788. 37343650 10.1016/j.jep.2023.116788

[fsn371746-bib-0027] Zeng, X. , L. Chen , and B. Zheng . 2024. “Extrusion and Chlorogenic Acid Treatment Increase the Ordered Structure and Resistant Starch Levels in Rice Starch With Amelioration of Gut Lipid Metabolism in Obese Rats.” Food & Function 15, no. 10: 5224–5237. 38623646 10.1039/d3fo05416k

[fsn371746-bib-0028] Zhang, Y. , S. Tu , X. Ji , et al. 2024. “Dubosiella Newyorkensis Modulates Immune Tolerance in Colitis via the L‐Lysine‐Activated AhR‐IDO1‐Kyn Pathway.” Nature Communications 15, no. 1: 1333. 10.1038/s41467-024-45636-x PMC10864277 38351003

[fsn371746-bib-0029] Zhang, Y. , X. Wang , J. Lin , et al. 2024. “A Microbial Metabolite Inhibits the HIF‐2α‐Ceramide Pathway to Mediate the Beneficial Effects of Time‐Restricted Feeding on MASH.” Cell Metabolism 36, no. 8: 1823–1838. 39079531 10.1016/j.cmet.2024.07.004

[fsn371746-bib-0030] Zhao, Y. , X. Liu , C. Ding , Y. Gu , and W. Liu . 2021. “Dihydromyricetin Reverses Thioacetamide‐Induced Liver Fibrosis Through Inhibiting NF‐κB‐Mediated Inflammation and TGF‐β1‐Regulated of PI3K/Akt Signaling Pathway.” Frontiers in Pharmacology 12: 783886. 34867416 10.3389/fphar.2021.783886 PMC8634482

[fsn371746-bib-0031] Zheng, X. J. , H. Xiao , Z. Zeng , et al. 2014. “Composition and Serum Antioxidation of the Main Flavonoids From Fermented Vine Tea (Ampelopsis Grossedentata).” Journal of Functional Foods 9: 290–294.

[fsn371746-bib-0032] Zhou, M. , J. Ma , M. Kang , et al. 2024. “Flavonoids, Gut Microbiota, and Host Lipid Metabolism.” Engineering in Life Sciences 24, no. 5: 2300065. 38708419 10.1002/elsc.202300065 PMC11065335

